# OA04 Schnitzler’s Syndrome: A Diagnostic Consideration in Evaluating Patients With Chronic Urticaria And Monoclonal Gammopathy

**DOI:** 10.1093/rap/rkae117.004

**Published:** 2024-11-05

**Authors:** Faria Alam Nikita, Helen Lachmann, Dipak Roy, Wendy Shingler, Lucy Coates

**Affiliations:** Tameside General Hospital, Greater Manchester, United Kingdom; Lambgates Health Centre, Greater Manchester, United Kingdom; Royal Free London Hospital, London, United Kingdom; Tameside General Hospital, Greater Manchester, United Kingdom; Tameside General Hospital, Greater Manchester, United Kingdom; Tameside General Hospital, Greater Manchester, United Kingdom

## Abstract

**Introduction:**

Schnitzler syndrome is a rare and under-recognised syndrome characterised by chronic urticaria, a monoclonal protein, and a number of other symptoms, including recurrent fever, arthralgia, fatigue, organomegaly- hepatosplenomegaly/lymphadenopathy and evidence of acute phase response. Biopsy of an involved area of the skin shows a neutrophilic infiltrate without evidence of vasculitis or haemorrhage. Underlying pathophysiology of this enigmatic syndrome remains unclear, partly because of its rarity, however, current evidence suggests this is an acquired auto-inflammatory syndrome with IL-1 being a key mediator. Recognition of this syndrome is critical since it is highly responsive to Anakinra^.^

**Case description:**

A 78 year old caucasian male, with no co-morbidities, initially presented to dermatologist with recurrent, pruritic urticarial hives, resistant to anti-histamines. Meanwhile, he developed acute illness with fever, rigor, fatigue, high inflammatory markers and was found to have anaemia requiring iron transfusion. Free light chains were present in urine and he was referred to haematology for possible myelo/lympho-proliferative disorder. Haemato-oncology work up included, CTTAP-showing axillary, cervical and iliac lymphadenopathy, splenomegaly and sclerotic area on iliac bone, lymph node biopsy- demonstrating low grade Non-Hodgkin’s Lymphoma, skeletal survey- suggesting degenerative changes and myeloma screen- concluding MGUS with monoclonal bands 2 IgM, 1 IgM. He was treated with Rituximab with no significant improvement, and therefore, was stopped and haematology adopted a policy of judicious monitoring.

After 3 years of persistent symptoms of fever, worsening fatigue, weight loss, pruritic urticarial rash, and persistently low Hb (92 g/L) and high acute phase reactants (CRP 197, ESR 112 at highest point), He was referred from haematology with a transient positive ANA, Rheumatoid factor, Anti-dsDNA and low complement levels (C4 0.04) to rheumatology. He denied any specific features of CTD or vasculitides. Skin biopsy revealed neutrophilic margination in dermis. Extensive rheumatology work up was performed which was normal and PET-CT revealed axillary lymphadenopathy, and repeat biopsy leading to a diagnosis of probable CLL along with NHL. Meanwhile, he was trialed on prednisolone with moderate response and subsequently on methotrexate as steroid sparing therapy with agreement from haemtology but clear diagnosis remained elusive.

He was also tested for hypo-complementemic urticarial vasculitis, a genetic condition and was referred to local immunology centre, genetics and National Amyloidosis Centre at Royal Free Hospital. When reviewed at the Royal Free Hospital, his clinical and laboratory findings rapidly led to a diagnosis of Schnitzler’s syndrome and he is about to start Anakinra.

**Discussion:**

On presentation to rheumatology after 3 years of onset of symptoms, the combination of clinical features of fever, fatigue and urticaria and biochemical features of persistent anaemia, leukocytosis and raised CRP, ESR, low complements, transiently positive ANA and Anti-dsDNA raised numerous possible diagnoses.

Considering the differential for a pyrexia of unknown origin (PUO), causes were systematically worked through and excluded including infection, malignancy (though recognizing his haematological findings), autoimmune disease, granulomatous disease, vasculitides and auto-inflammatory conditions. The patient was fully worked up with a thorough history, examination and investigations including autoimmune profile, C1 esterase inhibitor, IgG subtypes, ferritin, comprehensive infection screen, skeletal survey, PET scan, lymph node and skin biopsy were performed. The skin biopsy demonstrated a neutrophilic infiltrate, lymph node biopsy excluded granulomatous disease and demonstrated findings in keeping with a low grade B cell lymphoma and atypical CLL. Genetic analysis for possible Hypo-complementemic urticarial vasculitis was requested but remains outstanding. Immunology opinion was sought and liaison with both haematology and dermatology was maintained.

After recurrent inconclusive investigations at this stage, the patient was trialed on prednisolone to which he had moderate response (CRP halved and Hb returned to normal range), however, a few of the patient’s clinical features relapsed on attempt of dose reduction or withdrawal, therefore, the patient was offered methotrexate as immunosuppressant as well as steroid sparing agent but with no significant improvement of symptoms.

Finally, the patient was referred to the tertiary Amyloidosis Centre at Royal Free London Hospital, where a diagnosis of Schnitzler’s Syndrome was made and the patient is about to start Anakinra, IL-1 Antagonist, whilst being slowly weaned off steroid and Methotrexate

**Key learning points:**

• Schnitzler’s syndrome is a rare, late onset and acquired auto-immune condition which should be suspected in patients with chronic urticaria and monoclonal gammopathy and should be included, with other auto-inflammatory conditions, as a differential for pyrexia of unknown origin.

• Mean age of diagnosis is 50 years, men more affected than women, with average time required to reach diagnosis being 5 years from initial presentation and adding significant morbidities to the affected patients.

• Around 300 cases reported world-wide and 150 cases in Europe accounting for the rarity of the disease, therefore, it remains under-diagnosed. Prognosis is good if timely diagnosis is reached and treatment with Anakinra is established which is relatively well tolerated apart from injection site reactions and risk of neutropenic sepsis.

• 10-20% of the cases can develop a haematological malignancy including Waldenstrom Macroglobulinemia, IgM myeloma and Non-Hodgkin’s Lymphoma. The mean duration of developing this complication is 8 years from onset of symptoms. Interestingly, Anakinra offsets the development of the lympho-proliferative disorders, however, patients would require haemato-oncology follow up. AA amyloidosis can occur in untreated cases.

• With such rare condition, it is unlikely that general rheumatologist or haematologist would see more than one or two cases if any. When sensible investigations are not yielding diagnosis, it would be prudent to seek advice from centres of excellence to prevent delay in diagnosis.

• Strasbourg Criteria used for diagnosis-

• 2 Obligate criteria- Chronic urticarial rash & Monoclonal IgM or IgG

• Minor criteria- Recurrent fever, objective bone remodelling with or without bone pain, a neutrophilic dermal infiltrate on skin biopsy, leukocytosis and/or elevated CRP

• Definite diagnosis- both obligate criteria and at least two minor criteria if IgM / at least three minor criteria if IgG

• Probable diagnosis- Both obligate criteria and at least one minor criterion if IgM / at least two minor criteria if IgG

